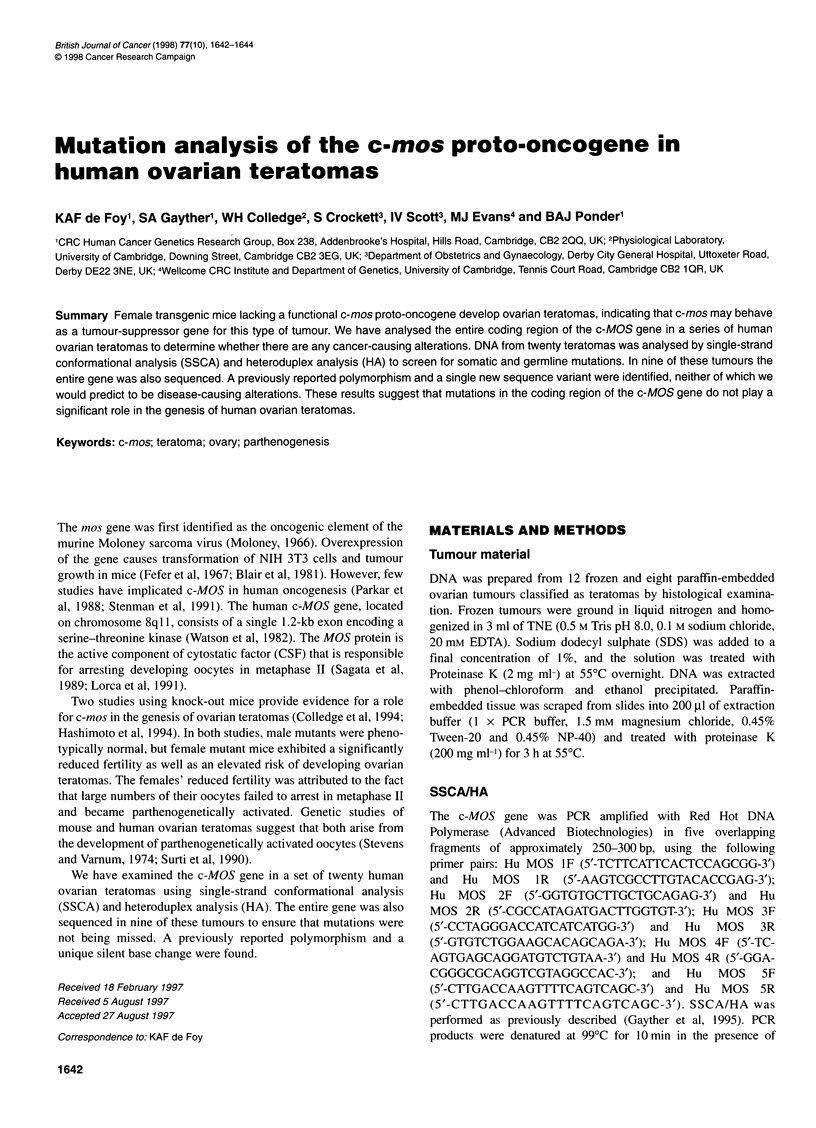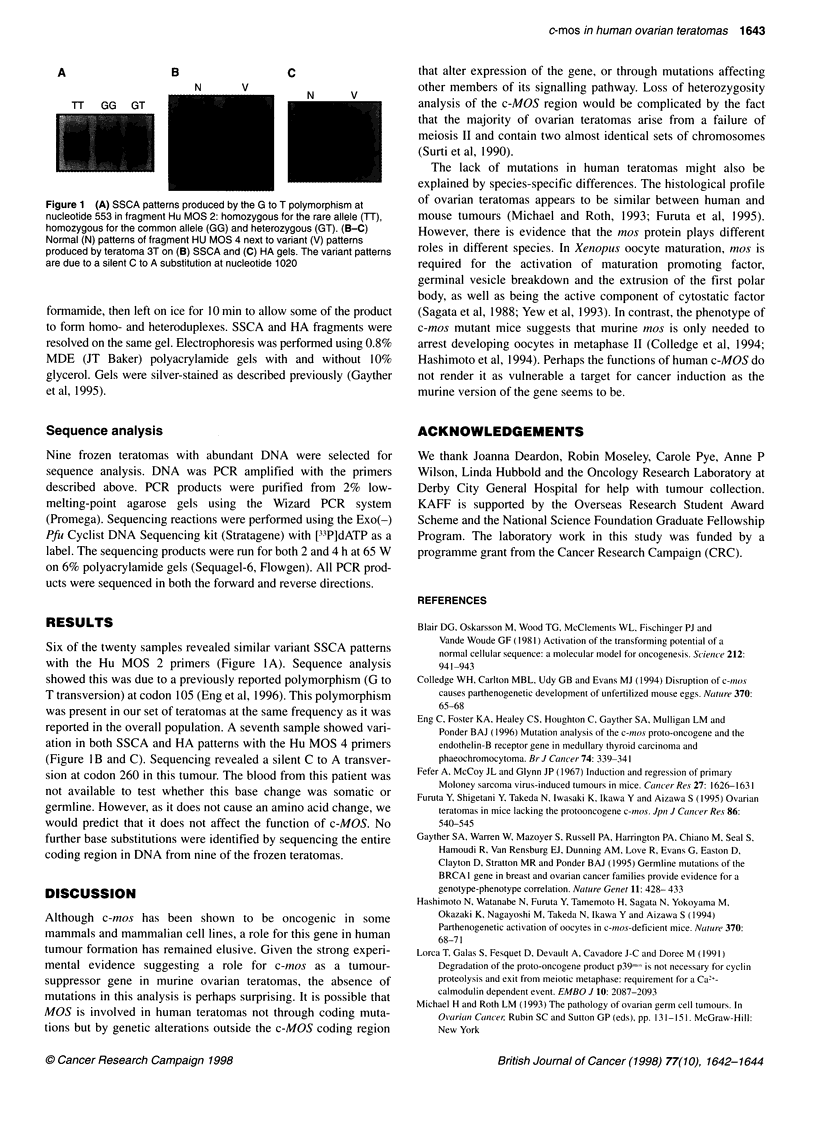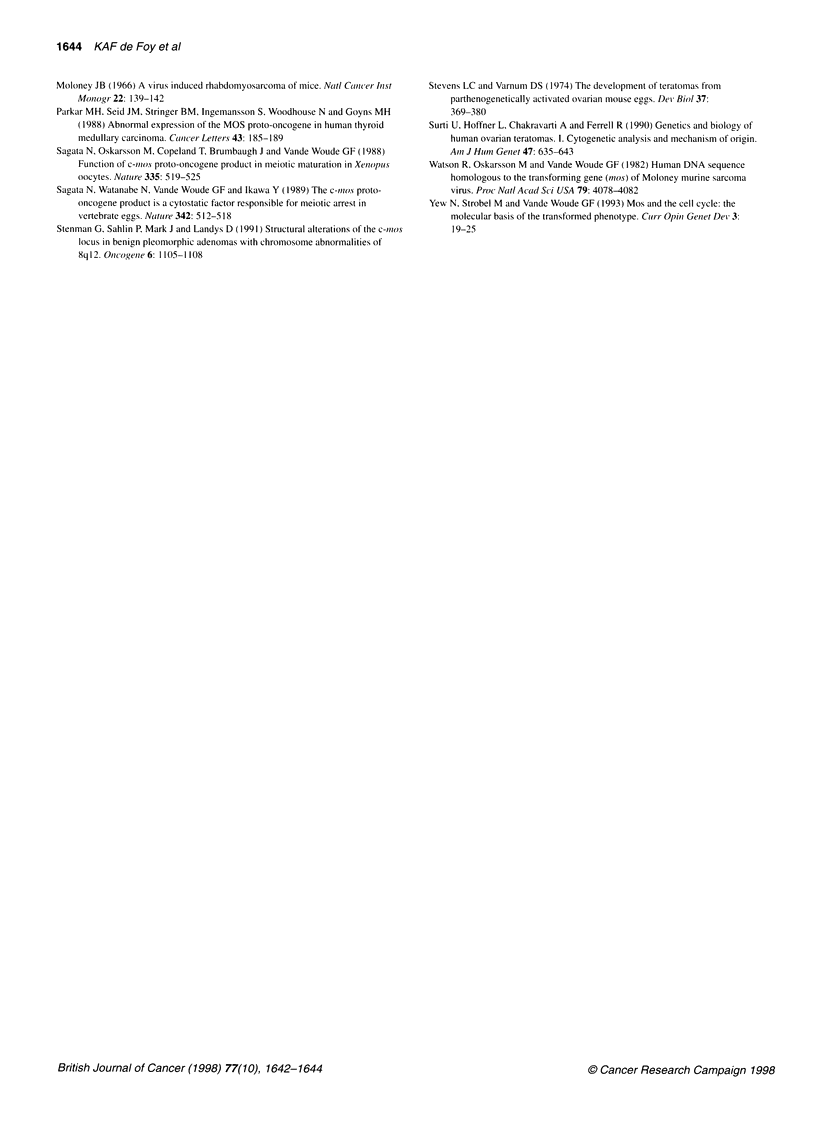# Mutation analysis of the c-mos proto-oncogene in human ovarian teratomas.

**DOI:** 10.1038/bjc.1998.269

**Published:** 1998-05

**Authors:** K. A. de Foy, S. A. Gayther, W. H. Colledge, S. Crockett, I. V. Scott, M. J. Evans, B. A. Ponder

**Affiliations:** CRC Human Cancer Genetics Research Group, Addenbrooke's Hospital, Cambridge, UK.

## Abstract

**Images:**


British Journal of Cancer (1998) 77(10), 1642–1644
© 1998 Cancer Research Campaign

### Mutation analysis of the c-*mos* proto-oncogene in human ovarian teratomas

KAF de Foy[1], SA Gayther[1], WH Colledge[2], S Crockett[3], IV Scott[3], MJ Evans[4] and BAJ Ponder[1]

[1]CRC Human Cancer Genetics Research Group, Box 238, Addenbrooke's Hospital, Hills Road, Cambridge, CB2 2QQ, UK; [2]Physiological Laboratory, University of Cambridge, Downing Street, Cambridge CB2 3EG, UK; [3]Department of Obstetrics and Gynaecology, Derby City General Hospital, Uttoxeter Road, Derby DE22 3NE, UK; [4]Wellcome CRC Institute and Department of Genetics, University of Cambridge, Tennis Court Road, Cambridge CB2 1QR, UK

**Summary** Female transgenic mice lacking a functional c-*mos* proto-oncogene develop ovarian teratomas, indicating that c-*mos* may behave as a tumour-suppressor gene for this type of tumour. We have analysed the entire coding region of the c-*MOS* gene in a series of human ovarian teratomas to determine whether there are any cancer-causing alterations. DNA from twenty teratomas was analysed by single-strand conformational analysis (SSCA) and heteroduplex analysis (HA) to screen for somatic and germline mutations. In nine of these tumours the entire gene was also sequenced. A previously reported polymorphism and a single new sequence variant were identified, neither of which we would predict to be disease-causing alterations. These results suggest that mutations in the coding region of the c-*MOS* gene do not play a significant role in the genesis of human ovarian teratomas.

**Keywords:** c-*mos*; teratoma; ovary; parthenogenesis

The *mos* gene was first identified as the oncogenic element of the murine Moloney sarcoma virus (Moloney, 1966). Overexpression of the gene causes transformation of NIH 3T3 cells and tumour growth in mice (Fefer et al, 1967; Blair et al, 1981). However, few studies have implicated c-*MOS* in human oncogenesis (Parkar et al, 1988; Stenman et al, 1991). The human c-*MOS* gene, located on chromosome 8q11, consists of a single 1.2-kb exon encoding a serine–threonine kinase (Watson et al, 1982). The *MOS* protein is the active component of cytostatic factor (CSF) that is responsible for arresting developing oocytes in metaphase II (Sagata et al, 1989; Lorca et al, 1991).

Two studies using knock-out mice provide evidence for a role for c-*mos* in the genesis of ovarian teratomas (Colledge et al, 1994; Hashimoto et al, 1994). In both studies, male mutants were phenotypically normal, but female mutant mice exhibited a significantly reduced fertility as well as an elevated risk of developing ovarian teratomas. The females' reduced fertility was attributed to the fact that large numbers of their oocytes failed to arrest in metaphase II and became parthenogenetically activated. Genetic studies of mouse and human ovarian teratomas suggest that both arise from the development of parthenogenetically activated oocytes (Stevens and Varnum, 1974; Surti et al, 1990).

We have examined the c-*MOS* gene in a set of twenty human ovarian teratomas using single-strand conformational analysis (SSCA) and heteroduplex analysis (HA). The entire gene was also sequenced in nine of these tumours to ensure that mutations were not being missed. A previously reported polymorphism and a unique silent base change were found.

*Received 18 February 1997*
*Received 5 August 1997*
*Accepted 27 August 1997*

*Correspondence to:* KAF de Foy

### MATERIALS AND METHODS

### Tumour material

DNA was prepared from 12 frozen and eight paraffin-embedded ovarian tumours classified as teratomas by histological examination. Frozen tumours were ground in liquid nitrogen and homogenized in 3 ml of TNE (0.5 M Tris pH 8.0, 0.1 M sodium chloride, 20 mM EDTA). Sodium dodecyl sulphate (SDS) was added to a final concentration of 1%, and the solution was treated with Proteinase K (2 mg $ml^{-}$) at 55°C overnight. DNA was extracted with phenol–chloroform and ethanol precipitated. Paraffin-embedded tissue was scraped from slides into 200 µl of extraction buffer (1 × PCR buffer, 1.5 mM magnesium chloride, 0.45% Tween-20 and 0.45% NP-40) and treated with proteinase K (200 mg $ml^{-1}$) for 3 h at 55°C.

### SSCA/HA

The c-*MOS* gene was PCR amplified with Red Hot DNA Polymerase (Advanced Biotechnologies) in five overlapping fragments of approximately 250–300 bp, using the following primer pairs: Hu MOS 1F (5′-TCTTCATTCACTCCAGCGG-3′) and Hu MOS 1R (5′-AAGTCGCCTTGTACACCGAG-3′); Hu MOS 2F (5′-GGTGTGCTTGCTGCAGAG-3′) and Hu MOS 2R (5′-CGCCATAGATGACTTGGTGT-3′); Hu MOS 3F (5′-CCTAGGGACCATCATCATGG-3′) and Hu MOS 3R (5′-GTGTCTGGAAGCACAGCAGA-3′); Hu MOS 4F (5′-TC-AGTGAGCAGGATGTCTGTAA-3′) and Hu MOS 4R (5′-GGA-CGGGCGCAGGTCGTAGGCCAC-3′); and Hu MOS 5F (5′-CTTGACCAAGTTTTCAGTCAGC-3′) and Hu MOS 5R (5′-CTTGACCAAGTTTTCAGTCAGC-3′). SSCA/HA was performed as previously described (Gayther et al, 1995). PCR products were denatured at 99°C for 10 min in the presence of

**Figure 1** **(A)** SSCA patterns produced by the G to T polymorphism at nucleotide 553 in fragment Hu MOS 2: homozygous for the rare allele (TT), homozygous for the common allele (GG) and heterozygous (GT). **(B–C)** Normal (N) patterns of fragment HU MOS 4 next to variant (V) patterns produced by teratoma 3T on **(B)** SSCA and **(C)** HA gels. The variant patterns are due to a silent C to A substitution at nucleotide 1020

formamide, then left on ice for 10 min to allow some of the product to form homo- and heteroduplexes. SSCA and HA fragments were resolved on the same gel. Electrophoresis was performed using 0.8% MDE (JT Baker) polyacrylamide gels with and without 10% glycerol. Gels were silver-stained as described previously (Gayther et al, 1995).

### Sequence analysis

Nine frozen teratomas with abundant DNA were selected for sequence analysis. DNA was PCR amplified with the primers described above. PCR products were purified from 2% low-melting-point agarose gels using the Wizard PCR system (Promega). Sequencing reactions were performed using the Exo(–) *Pfu* Cyclist DNA Sequencing kit (Stratagene) with [$^{33}$P]dATP as a label. The sequencing products were run for both 2 and 4 h at 65 W on 6% polyacrylamide gels (Sequagel-6, Flowgen). All PCR products were sequenced in both the forward and reverse directions.

### RESULTS

Six of the twenty samples revealed similar variant SSCA patterns with the Hu MOS 2 primers (Figure 1A). Sequence analysis showed this was due to a previously reported polymorphism (G to T transversion) at codon 105 (Eng et al, 1996). This polymorphism was present in our set of teratomas at the same frequency as it was reported in the overall population. A seventh sample showed variation in both SSCA and HA patterns with the Hu MOS 4 primers (Figure 1B and C). Sequencing revealed a silent C to A transversion at codon 260 in this tumour. The blood from this patient was not available to test whether this base change was somatic or germline. However, as it does not cause an amino acid change, we would predict that it does not affect the function of c-*MOS*. No further base substitutions were identified by sequencing the entire coding region in DNA from nine of the frozen teratomas.

### DISCUSSION

Although c-*mos* has been shown to be oncogenic in some mammals and mammalian cell lines, a role for this gene in human tumour formation has remained elusive. Given the strong experimental evidence suggesting a role for c-*mos* as a tumour-suppressor gene in murine ovarian teratomas, the absence of mutations in this analysis is perhaps surprising. It is possible that *MOS* is involved in human teratomas not through coding mutations but by genetic alterations outside the c-*MOS* coding region that alter expression of the gene, or through mutations affecting other members of its signalling pathway. Loss of heterozygosity analysis of the c-*MOS* region would be complicated by the fact that the majority of ovarian teratomas arise from a failure of meiosis II and contain two almost identical sets of chromosomes (Surti et al, 1990).

The lack of mutations in human teratomas might also be explained by species-specific differences. The histological profile of ovarian teratomas appears to be similar between human and mouse tumours (Michael and Roth, 1993; Furuta et al, 1995). However, there is evidence that the *mos* protein plays different roles in different species. In *Xenopus* oocyte maturation, *mos* is required for the activation of maturation promoting factor, germinal vesicle breakdown and the extrusion of the first polar body, as well as being the active component of cytostatic factor (Sagata et al, 1988; Yew et al, 1993). In contrast, the phenotype of c-*mos* mutant mice suggests that murine *mos* is only needed to arrest developing oocytes in metaphase II (Colledge et al, 1994; Hashimoto et al, 1994). Perhaps the functions of human c-*MOS* do not render it as vulnerable a target for cancer induction as the murine version of the gene seems to be.

### ACKNOWLEDGEMENTS

We thank Joanna Deardon, Robin Moseley, Carole Pye, Anne P Wilson, Linda Hubbold and the Oncology Research Laboratory at Derby City General Hospital for help with tumour collection. KAFF is supported by the Overseas Research Student Award Scheme and the National Science Foundation Graduate Fellowship Program. The laboratory work in this study was funded by a programme grant from the Cancer Research Campaign (CRC).

#### REFERENCES

Blair DG, Oskarsson M, Wood TG, McClements WL, Fischinger PJ and Vande Woude GF (1981) Activation of the transforming potential of a normal cellular sequence: a molecular model for oncogenesis. *Science* **212**: 941–943

Colledge WH, Carlton MBL, Udy GB and Evans MJ (1994) Disruption of c-*mos* causes parthenogenetic development of unfertilized mouse eggs. *Nature* **370**: 65–68

Eng C, Foster KA, Healey CS, Houghton C, Gayther SA, Mulligan LM and Ponder BAJ (1996) Mutation analysis of the c-*mos* proto-oncogene and the endothelin-B receptor gene in medullary thyroid carcinoma and phaeochromocytoma. *Br J Cancer* **74**: 339–341

Fefer A, McCoy JL and Glynn JP (1967) Induction and regression of primary Moloney sarcoma virus-induced tumours in mice. *Cancer Res* **27**: 1626–1631

Furuta Y, Shigetani Y, Takeda N, Iwasaki K, Ikawa Y and Aizawa S (1995) Ovarian teratomas in mice lacking the protooncogene c-*mos*. *Jpn J Cancer Res* **86**: 540–545

Gayther SA, Warren W, Mazoyer S, Russell PA, Harrington PA, Chiano M, Seal S, Hamoudi R, Van Rensburg EJ, Dunning AM, Love R, Evans G, Easton D, Clayton D, Stratton MR and Ponder BAJ (1995) Germline mutations of the BRCA1 gene in breast and ovarian cancer families provide evidence for a genotype-phenotype correlation. *Nature Genet* **11**: 428– 433

Hashimoto N, Watanabe N, Furuta Y, Tamemoto H, Sagata N, Yokoyama M, Okazaki K, Nagayoshi M, Takeda N, Ikawa Y and Aizawa S (1994) Parthenogenetic activation of oocytes in c-*mos*-deficient mice. *Nature* **370**: 68–71

Lorca T, Galas S, Fesquet D, Devault A, Cavadore J-C and Doree M (1991) Degradation of the proto-oncogene product p39$^{mos}$ is not necessary for cyclin proteolysis and exit from meiotic metaphase: requirement for a $Ca^{2+}$-calmodulin dependent event. *EMBO J* **10**: 2087–2093

Michael H and Roth LM (1993) The pathology of ovarian germ cell tumours. In *Ovarian Cancer*, Rubin SC and Sutton GP (eds), pp. 131–151. McGraw-Hill: New York

© Cancer Research Campaign 1998

Moloney JB (1966) A virus induced rhabdomyosarcoma of mice. *Natl Cancer Inst Monogr* **22**: 139–142

Parkar MH, Seid JM, Stringer BM, Ingemansson S, Woodhouse N and Goyns MH (1988) Abnormal expression of the MOS proto-oncogene in human thyroid medullary carcinoma. *Cancer Letters* **43**: 185–189

Sagata N, Oskarsson M, Copeland T, Brumbaugh J and Vande Woude GF (1988) Function of c-*mos* proto-oncogene product in meiotic maturation in *Xenopus* oocytes. *Nature* **335**: 519–525

Sagata N, Watanabe N, Vande Woude GF and Ikawa Y (1989) The c-*mos* proto-oncogene product is a cytostatic factor responsible for meiotic arrest in vertebrate eggs. *Nature* **342**: 512–518

Stenman G, Sahlin P, Mark J and Landys D (1991) Structural alterations of the c-*mos* locus in benign pleomorphic adenomas with chromosome abnormalities of 8q12. *Oncogene* **6**: 1105–1108

Stevens LC and Varnum DS (1974) The development of teratomas from parthenogenetically activated ovarian mouse eggs. *Dev Biol* **37**: 369–380

Surti U, Hoffner L, Chakravarti A and Ferrell R (1990) Genetics and biology of human ovarian teratomas. I. Cytogenetic analysis and mechanism of origin. *Am J Hum Genet* **47**: 635–643

Watson R, Oskarsson M and Vande Woude GF (1982) Human DNA sequence homologous to the transforming gene (*mos*) of Moloney murine sarcoma virus. *Proc Natl Acad Sci USA* **79**: 4078–4082

Yew N, Strobel M and Vande Woude GF (1993) Mos and the cell cycle: the molecular basis of the transformed phenotype. *Curr Opin Genet Dev* **3**: 19–25

*© Cancer Research Campaign 1998*